# A Bibliometric Analysis of Pulmonary Alveolar Proteinosis From 2001 to 2021

**DOI:** 10.3389/fmed.2022.846480

**Published:** 2022-03-22

**Authors:** Shixu Liu, Xiangning Cui, Kun Xia, Dandan Wang, Jing Han, Xiaoyan Yao, Xiaohong Liu, Lingjie Bian, Jinzhi Zhang, Guangxi Li

**Affiliations:** ^1^Guanganmen Hospital, China Academy of Chinese Medical Sciences, Beijing, China; ^2^Graduate School of China Academy of Chinese Medical Sciences, Beijing, China; ^3^Affilated Hospital of Weifang Medical University, Weifang, China; ^4^Dongzhimen Hospital, Beijing University of Chinese Medicine, Beijing, China

**Keywords:** pulmonary alveolar proteinosis (PAP), bibliometric analysis, CiteSpace, VOSviewer, autoimmune pulmonary alveolar proteinosis (aPAP), alveolar macrophage, granulocyte-macrophage colony-stimulating factor (GM-CSF)

## Abstract

**Background:**

Pulmonary alveolar proteinosis (PAP) is a rare syndrome first described by Rosen et al. in 1958. Despite our considerably evolved understanding of PAP over the past decades, no bibliometric studies have been reported on this field. We aimed to analyze and visualize the research hotspots and current trends of the PAP research field using a bibliometric analysis to help understand the future development of basic and clinical research.

**Methods:**

The literature regarding PAP was culled from the Web of Science Core Collection (WoSCC) database. Data were extracted from the relevant articles and visually analyzed using CiteSpace and VOSviewer software.

**Results:**

Nine hundred and nine qualifying articles were included in the analysis. Publications regarding PAP increased over time. These articles mainly come from 407 institutions of 57 countries. The leading countries were the USA and Japan. University of Cincinnati (USA) and Niigata University (Japan) featured the highest number of publications among all institutions. Bruce C Trapnell exerts a significant publication impact and has made the most outstanding contributions in the field of PAP. *American Journal of Physiology-Lung Cellular and Molecular Physiology* was the journal with the most publications, and *American Journal of Respiratory and Critical Care Medicine* was the most commonly cited journal. All the top 5 co-cited journals belong to Q1. Keyword citation bursts revealed that inflammation, deficiency, tissue resident macrophage, classification, autoimmune pulmonary alveolar proteinosis, sarcoidosis, gm csf, high resolution ct, and fetal monocyte were the emerging research hotspots.

**Conclusion:**

Research on PAP is prosperous. International cooperation is also expected to deepen and strengthen in the future. Our results indicated that the etiology and pathogenesis of PAP, current and emerging therapies, especially the novel pathogenesis-based options will remain research hotspots in the future.

## Introduction

Pulmonary alveolar proteinosis (PAP) is a rare syndrome that was first described in 1958 by Rosen et al. (1). Recently, the prevalence of PAP has been estimated to be 6.87 per million in the general population, without gender predilection (2); the evidence backing global variation in the epidemiology of PAP is insubstantial (3). PAP is characterized by altered surfactant homeostasis and resultant accumulation of lipoproteinaceous material in pulmonary alveoli and alveolar macrophages (AMs) (4, 5). Pathologically, PAP is a heterogeneous group of diseases that result from either poor surfactant clearance or abnormal surfactant production due to AMs dysfunction (3). Granulocyte-macrophage colony-stimulating factor (GM-CSF) plays a pivotal role in the terminal differentiation of AMs, which is crucial for AMs regulating innate immunity and surfactant catabolism (6).

The typical physiological consequence of PAP is impaired gas exchange, resulting in progressive dyspnoea, hypoxemia, or even respiratory failure and death (5). There is currently no cure for PAP, but it can be treated. By tradition, the standard gold therapy of PAP has been whole-lung lavage (WLL), where large quantities of saline are instilled into the lungs to clear the abnormal surfactant. Other therapeutic strategies have been investigated by targeting AMs with GM-CSF augmentation or reducing the levels of GM-CSF autoantibodies with CD20 antibody rituximab and experimental plasmapheresis. New treatment modalities, such as statins, pioglitazone, lung transplantation, and pulmonary macrophage transplantation, are promising approaches requiring further research. Notwithstanding significant progress in our understanding of PAP over the past decades, comprehensive reports that can help scholars obtain an intuitive overview and disclose trends in the PAP research field are still absent.

Bibliometric analysis is a novel scientific method used to analyze large amounts of heterogeneous literature (7). Combining visualizing processing tools like CiteSpace (8) and VOSviewer (9) helps comprehend the knowledge structure and explore developmental trends. Bibliometric analysis can not only evaluate the contributions of various authors, institutions, countries/regions, and journals, but also can predict the research hotspots and trends of a specific research field, laying the foundation for the development of future study (10, 11). However, there is still a lack of bibliometric analysis in PAP research. The present study aimed to explore the hotspots and developmental trends of PAP by analyzing historic achievements from 2001 to 2021 to provide new visions for future researchers, especially for those who have an interest but are new to this field.

## Materials and Methods

### Data Collection

**Figure 1:**
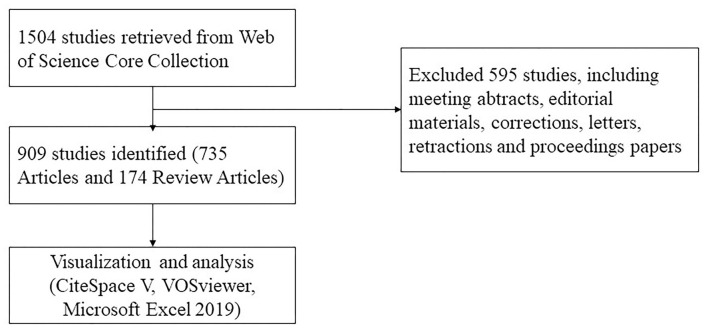
Flowchart of literature selection.

A literature search was conducted using the Web of Science Core Collection database on December 11, 2021. Editions selected “Science Citation Index Expanded (SCI-EXPANDED).” The search strategy was set to TS = (“pulmonary alveolar proteinosis”) AND Language = English from 2001 to 2021. A total of 1,504 articles were retrieved, 595 irrelevant articles, including meeting abstracts, editorial materials, corrections, letters, retractions, and proceedings papers, were excluded. A total of 909 papers were exported in the form of full records and cited references and saved in download_txt format within 1 day (Figure 1).

### Data Analysis

Records retrieved from Web of Science Core Collection were imported to Microsoft Excel 2019, using CiteSpace and VOSviewer to analyze and visualize retrieval results. In the atlas, nodes represent countries/regions, institutions, authors, co-cited literature, journals, and keywords; links between nodes usually mean cooperation or co-citation relationships; colors of the nodes and links alter over time.

CiteSpace version 5.8.R3 (Drexel University, Philadelphia, PA, USA) was used to draw the maps of country/region and institutional cooperation, literature, and journal co-citation. CiteSpace is a tool for progressive knowledge domain visualization developed by Chen (12). It is particularly beneficial to visualize and analyze trends and patterns in scientific literature. The primary objective of knowledge domain visualization is to uncover critical points in the development of the domain. CiteSpace provides a visual aid that portrays research hotspots and evolution processes intuitively and predicts the developmental trends of the research field (8, 13).

VOSviewer version 1.6.17 (Leiden University, Leiden, Netherlands) was used to analyze and visualize the author's cooperation and keyword co-occurrence. VOSviewer is software for constructing and viewing bibliometric networks. Unlike the conventional bibliometric tools, VOSviewer concentrates on the graphic representation of bibliometric networks. Its most prominent feature is displaying large bibliometrics in an easy-to-explain way (9).

## Results

### The Trend of Publication Outputs

**Figure 2:**
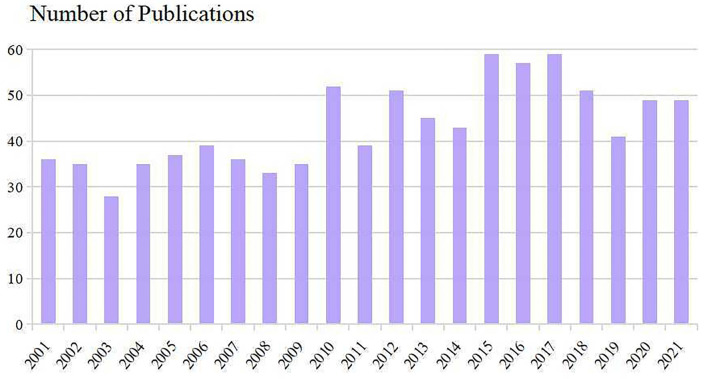
Trends of PAP publications over the past 20 years.

The number of publications and times cited in each period reflects the research trends in this field. As shown in Figure 2, the number of studies concerning PAP manifested an overall upward trend. Nine hundred and nine included scientific literature (including 735 Articles and 174 Review Articles) with the times cited of 28,550, the average citations of 31.41 per item, and an h-index of 81. 2015–2017 were the most productive period with a total of 175 articles. Despite reducing publications since 2018, Results showed that research on PAP remains at a relatively high level.

### Contribution of Countries/Regions and Institutions

**Figure 3:**
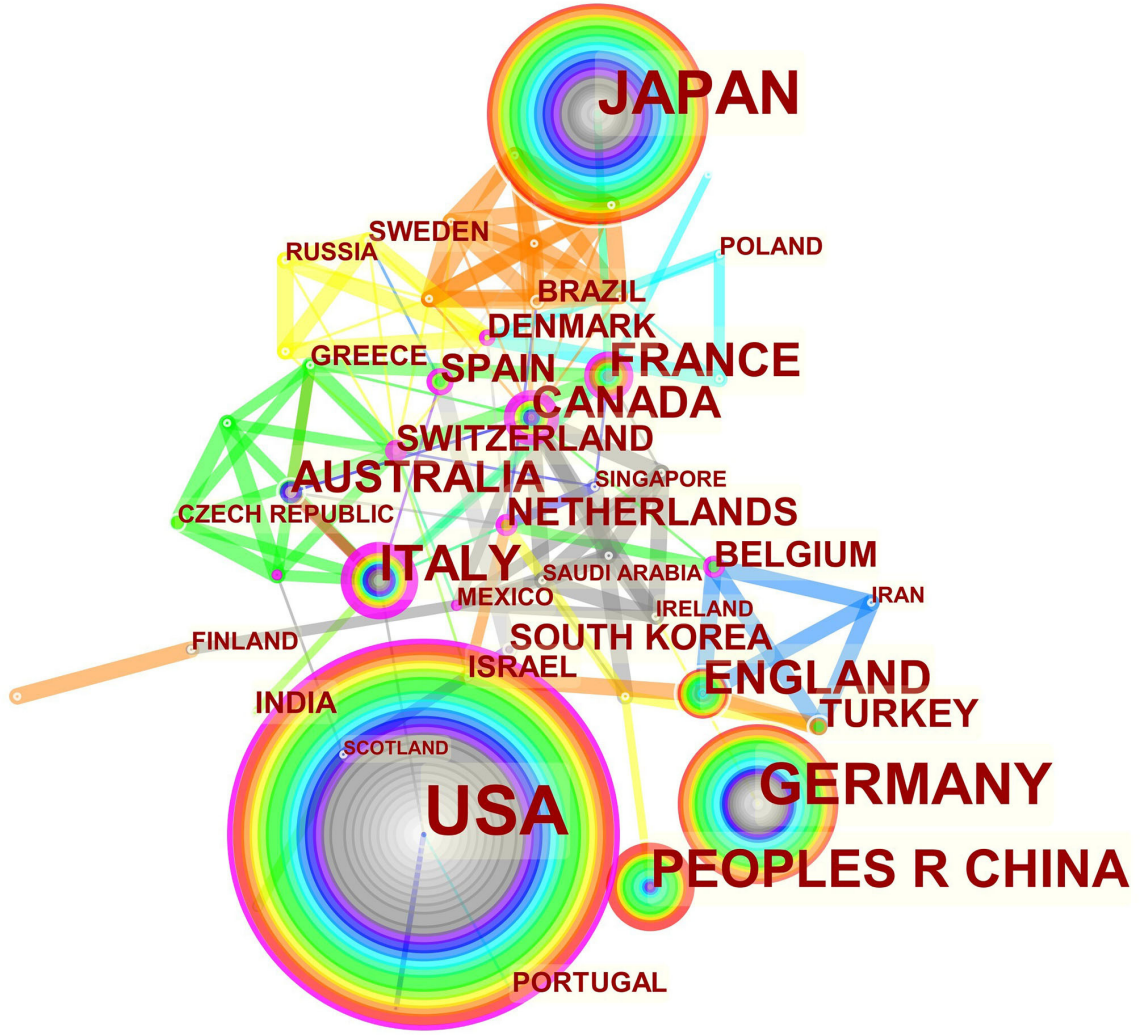
Distribution of publications from different countries/regions.

**Figure 4:**
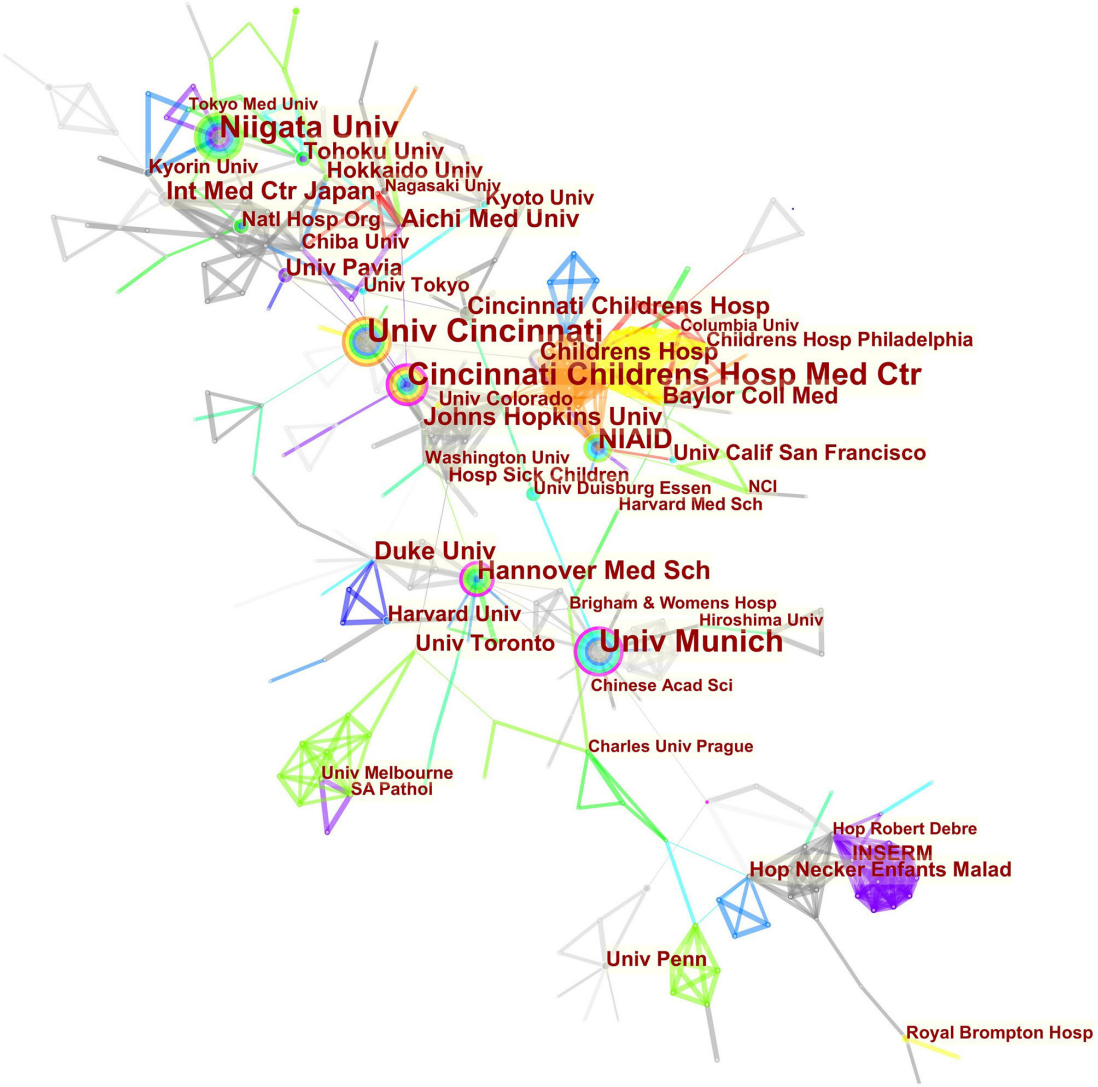
Distribution of publications from different institutions.

The maps of intercountry/regional cooperation (Figure 3, N = 57, E = 131) and inter-institutional cooperation (Figure 4, N = 473, E = 789) were generated using CiteSpace. Researchers from 407 institutions in 57 countries/regions contributed to publications on PAP between 2001 and 2021. As shown in these figures, each node represents a country or institution; the node's size is proportional to the publications. The lines between the nodes stand for cooperation between countries or institutions; the wider the lines, the closer the cooperation. Tables 1, 2 list the top 5 countries and institutions in this field. The largest contributor was the United States (*n* = 382, 42.0%), followed by Japan (*n* = 160, 17.6%), Germany (*n* = 116, 12.8%), China (*n* = 74, 8.1%), and Italy (*n* = 56, 6.2%). The publications from the two highest-ranked countries were nearly two-thirds of the total. University of Cincinnati (Cincinnati, Ohio, USA) was the most authoritative institution in the PAP research field, followed by Niigata University (Niigata, Niigata, Japan) and Cincinnati Children's Hospital Medical Center (Cincinnati, Ohio, USA). Countries such as Switzerland, Italy, and Canada, demonstrated a high degree of centrality, as indicated by the thickness of the purple rings in Figure 3. It is a measure associated with the transformative potential of a scientific contribution. Such nodes tend to bridge different stages of the development of a scientific field (14). There was active cooperation among countries and institutions, including Switzerland, Australia, Italy, Niigata University, Tohoku University, Hannover Medical School, and the University of Toronto. However, most countries/regions and institutions were dispersed and lacked intensive cooperation.

**Table 1:** The top 5 countries for publications and centrality in PAP research.

**No**.	**Country**	**Count (%)**	**Country**	**Centrality**
1	USA	382 (42.0%)	Switzerland	0.33
2	Japan	160 (17.6%)	Italy	0.31
3	Germany	116 (12.8%)	Canada	0.31
4	China	74 (8.1%)	Netherlands	0.2
5	Italy	56 (6.2%)	France	0.17

**Table 2:** The top 5 institutions for publications and centrality in PAP research.

**No**.	**Institution**	**Count (%)**	**Institution**	**Centrality**
1	Univ Cincinnati	36 (4.0%)	Cincinnati Childrens Hosp Med Ctr	0.26
2	Niigata Univ	33 (3.6%)	Hannover Med Sch	0.22
3	Cincinnati Childrens Hosp Med Ctr	32 (3.5%)	Univ Munich	0.21
4	Univ Munich	30 (3.3%)	Hop Enfants Armand Trousseau	0.15
5	NIAID	19 (2.1%)	Childrens Hosp Pittsburgh	0.13

### Authors and Co-cited Authors

Table 3 lists the top 5 most active authors and co-cited authors. BC Trapnell had the highest number of published papers (40, 4.4%). Among the top 5 authors, K Nakata (0.05) and Y Inoue (0.05) have high centralities, which implicates that these two authors significantly impact each other's work and studies from other groups.

**Table 3:** The top 10 authors and co-cited authors in PAP research.

**No**.	**Author**	**Count (%)**	**Centrality**	**Co-cited author**	**Citation**	**Centrality**
1	BC Trapnell	40 (4.4%)	0.03	JF Seymour	503	0
2	K Nakata	39 (4.3%)	0.05	BC Trapnell	405	0
3	T Suzuki	21 (2.3%)	0.04	LM Nogee	377	0
4	M Griese	19 (2.1%)	0	T Kitamura	352	0
5	Y Inoue	18 (2.0%)	0.05	T Suzuki	299	0

Co-cited authors are two or more authors cited simultaneously, and these authors form a co-citation relationship. Among all the co-cited authors, four had a citation frequency of more than 300 times. JF Seymour (503) was the most frequently cited author, followed by BC Trapnell (405).

**Figure 5:**
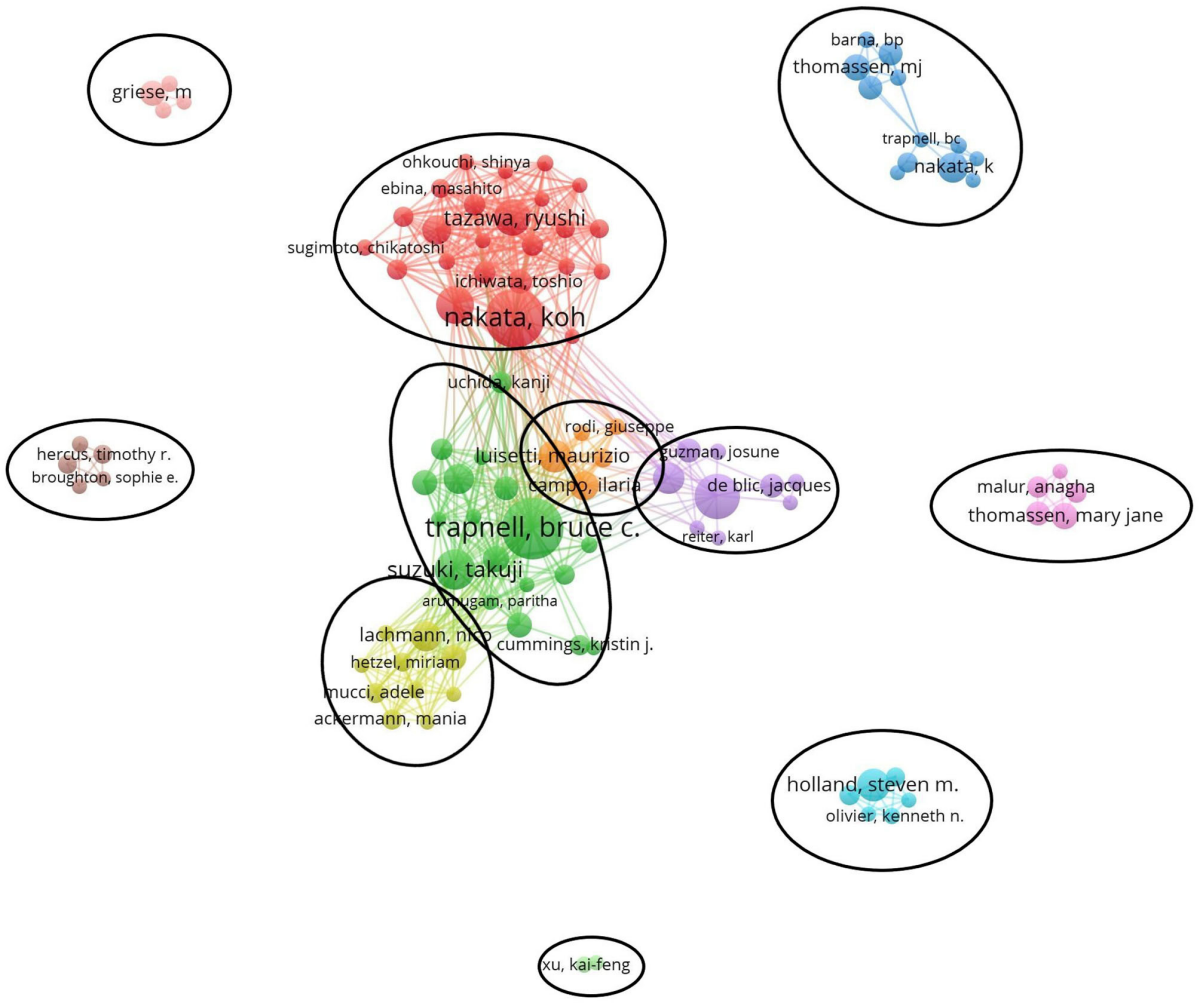
The active authors in the PAP research field: there are 13 clusters.

Figure 5 generated by VOSviewer presented the collaborative relationships and found 11 cooperation teams. Each node symbolizes an author, with larger nodes indicating more publications. Wider lines represent the closer connection between authors. The connection network of different colors manifests the cooperation cluster among authors in PAP research. As shown in Figure 5, there was an obvious connection network between different authors, for example, BC Trapnell, T Suzuki, K Uchida, and K Nakata, R Tazawa, T Ichiwata.

### Journals and Co-cited Journals

There were 398 academic journals related to PAP. The journal *American Journal of Physiology-Lung Cellular and Molecular Physiology* had the highest number of outputs (25, 2.8%). The *American Journal of Respiratory and Critical Care Medicine* ranked second (24, 2.6%) with the highest impact factor (IF = 16.671) among the top 10 academic journals. It can be seen in Table 4 that 80% of journals belong to Q1 and Q2 according to the journal citation reports (JCR) in 2020.

**Table 4:** The top 10 journals and co-cited journals related to PAP.

**No**.	**Journal**	**Count (%)**	**IF (2020)**	**JCR**	**Co-cited Journal**	**Citation**	**IF (2020)**	**JCR**
1	Am J Physiol Lung Cell Mol Physiol	25(2.8%)	5.464	Q2	Am J resp Crit Care	2,565	21.4	Q1
2	Am J resp Crit Care	24(2.6%)	21.4	Q1	New Engl J Med	1,525	91.245	Q1
3	Chest	24(2.6%)	9.410	Q1	Chest	1,519	9.410	Q1
4	Respirology	21(2.3%)	6.424	Q2	Blood	1,374	22.113	Q1
5	Orphanet J Rare Dis	17(1.9%)	4.123	Q2	Eur Respir J	1,008	16.671	Q1
6	Eur Respir J	16(1.8%)	16.671	Q1	J Immunol	997	5.422	Q2
7	Intern Med	14(1.5%)	1.271	Q4	J Biol Chem	976	5.157	Q2
8	BMC Pulm Med	13(1.4%)	3.317	Q3	Am J Physiol Lung Cell Mol Physiol	969	5.422	Q2
9	J Immunol	13(1.4%)	5.422	Q2	J Exp Med	957	14.307	Q1
10	Respir Res	13(1.4%)	5.631	Q2	P Natl Acad Sci USA	873	11.205	Q1

The influence of academic journals relies on the number of times they are co-cited, which shows whether the journal has notable influence in a domain. Among the top 10 co-cited journals, five journals have been cited more than 1,000 times. As shown in Table 4, the *American Journal of Respiratory and Critical Care Medicine* had the highest number of citations (2,565), followed by *The New England Journal of Medicine (NEJM)* (1,525), *Chest* (1,519), *Blood* (1,374), and *European Respiratory Journal* (1008). All the co-cited journals were distributed in the Q1 and Q2 region, and the *NEJM* had the highest impact factor (IF = 91.245).

### Co-cited References and References Burst

**Figure 6:**
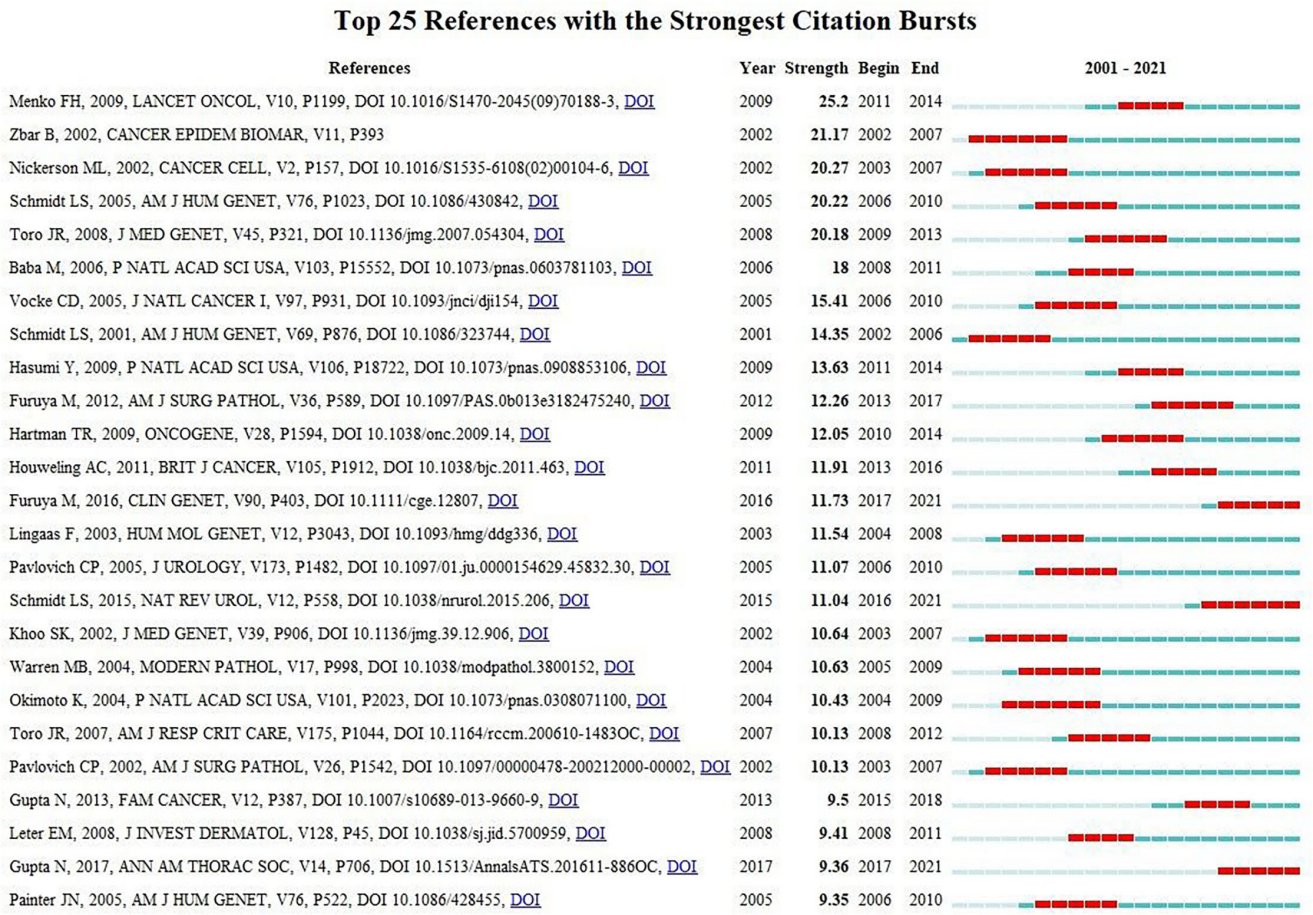
25 references with the strongest citation bursts related to PAP.

Co-citation means that two or more articles are cited together by at least one later publication. It is a measurement to quantify the degree of relationship between articles. Among the 24,740 cited references retrieved, Table 5 demonstrates the 10 most often cited references, of which Bruce C Trapnell's article published in *NEJM* in 2003 ranks the first. Figure 6 reveals that the first burst of co-cited reference began in 2001. The majority of them have been cited frequently during 2001–2021, which suggests that the research related to PAP may continue to be flourishing in the future.

**Table 5:** The top 10 co-cited references related to PAP.

**No**.	**References**	**Author**	**Year**	**Journal**	**Citation**	**References**
1	Pulmonary alveolar proteinosis	BC Trapnell	2003	New Engl J Med	277	(15)
2	Pulmonary alveolar proteinosis: progress in the first 44 years	JF Seymour	2002	Am J Resp Crit Care	251	(16)
3	Idiopathic pulmonary alveolar proteinosis as an autoimmune disease with neutralizing antibody against granulocyte/macrophage colony-stimulating factor	T Kitamura	1999	J Exp Med	246	(17)
4	Pulmonary alveolar proteinosis	SH Rosen	1958	New Engl J Med	201	(1)
5	Involvement of granulocyte-macrophage colony-stimulating factor in pulmonary homeostasis	G Dranoff	1994	Science	167	(18)
6	Characteristics of a large cohort of patients with autoimmune pulmonary alveolar proteinosis in Japan	Y Inoue	2008	Am J Resp Crit Care	139	(19)
7	Granulocyte/macrophage colony-stimulating factor-deficient mice show no major perturbation of hematopoiesis but develop a characteristic pulmonary pathology	E Stanley	1994	P Natl Acad Sci Usa	135	(20)
8	GM-CSF regulates alveolar macrophage differentiation and innate immunity in the lung through PU. 1	Y Shibata	2001	Immunity	111	(6)
9	High-affinity autoantibodies specifically eliminate granulocyte-macrophage colony-stimulating factor activity in the lungs of patients with idiopathic pulmonary alveolar proteinosis	K Uchida	2004	Blood	108	(21)
10	Familial pulmonary alveolar proteinosis caused by mutations in *CSF2RA*	T Suzuki	2008	J Exp Med	101	(22)

### The Analysis of Hotspots and Frontiers

Keywords sum up research topics and essences. High-frequency keywords are usually the dominant research direction in this field. Keyword co-occurrence analysis helps us determine research hotspots and predict research trends in a specific field (12). According to Table 6, excluding pulmonary alveolar proteinosis (476), The top 10 keywords with the highest frequency in 2001–2021 are colony-stimulating factor (258), disease (122), gm-csf (119), lung (95), diagnosis (67), expression (64), surfactant (23), deficient mice (24), therapy (25). Among these keywords, GM-CSF (including colony-stimulating factor and gm-csf) appeared over 400 times, indicating that it was the topical issue in the study of PAP.

**Table 6:** Top 20 keywords related to PAP.

**Rank**	**Keywords**	**Count**	**Rank**	**Keywords**	**Count**
1	pulmonary alveolar proteinosis	476	11	pulmonary surfactant	51
2	colony-stimulating factor	258	12	autoantibodies	50
3	disease	122	13	whole-lung lavage	50
4	gm-csf	119	14	macrophages	49
5	lung	95	15	mice	49
6	diagnosis	67	16	bronchoalveolar lavage	45
7	expression	64	17	mutations	43
8	surfactant	58	18	children	39
9	deficient mice	53	19	gene	39
10	therapy	52	20	inflammation	39

**Figure 7:**
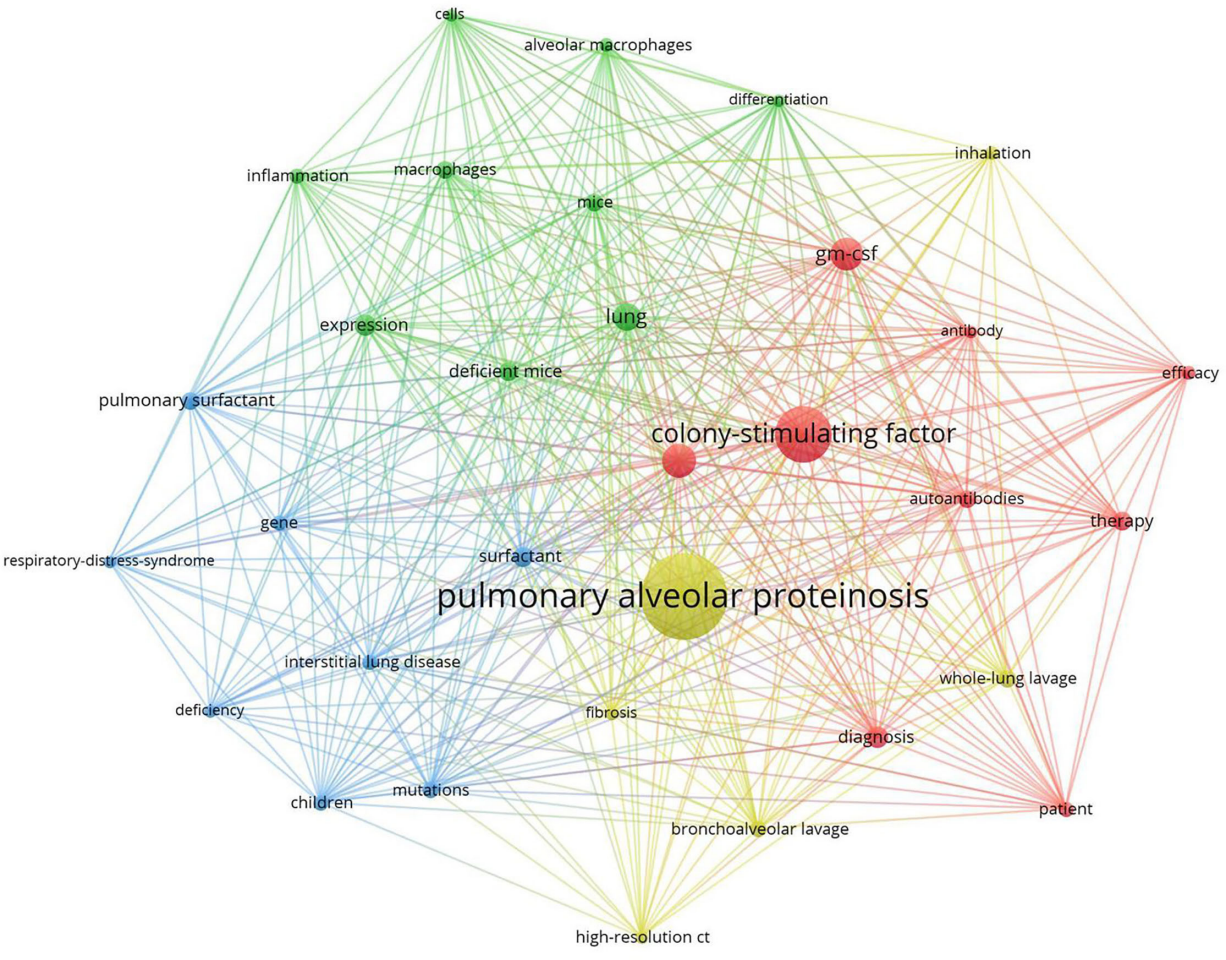
Keywords clustering analysis of the PAP research.

Clustered keywords reflect structures of knowledge in related study fields. We used VOSviewer software for this procedure. Nodes and labels form a unit, and units of different colors constitute different clusters. As shown in Figure 7, there are red, green, blue, and yellow clusters, representing four research directions. The main keywords of the red cluster are gm-csf, autoantibodies, diagnosis, therapy, efficacy, patient. The green cluster includes alveolar macrophages, cells, deficient mice, differentiation, expression, inflammation. Blue cluster mainly includes children, mutations, gene, deficiency, interstitial lung disease, pulmonary surfactant, respiratory distress syndrome, and of the yellow cluster are bronchoalveolar lavage, whole-lung lavage, high-resolution ct, inhalation, fibrosis.

Strong citation bursts can disclose hot words at the frontier of research. Table 7 cataloged the top 20 keywords with the strongest citation bursts. The top five hotspots were: pulmonary surfactant (11.69), respiratory distress syndrome (9.53), congenital alveolar proteinosis (6.38), surfactant protein b (5.95), deficient mice (5.23). Subsequently, keywords such as “inflammation,” “deficiency,” “tissue resident macrophage,” “classification,” “autoimmune pulmonary alveolar proteinosis,” “sarcoidosis,” “gm csf,” “high-resolution ct,” and “fetal monocyte” appeared frequently in the last 5 years (Table 8). It indicates that the early stage of PAP research was focused on surfactant metabolism and the etiology and pathogenesis of different classifications of PAP. Recently, the efficacy of emerging pathogenesis-based therapies (such as PMT) has addressed our attention. Autoimmune PAP will remain research hotspots in the coming years.

**Table 7:** Top 20 keywords with the strongest citation bursts.

**Keywords**	**Strength**	**Begin**	**End**	**2001–2021**
pulmonary surfactant	11.69	2001	2021	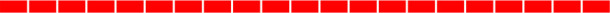
respiratory distress syndrome	9.53	2001	2021	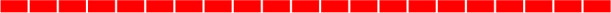
congenital alveolar proteinosis	6.38	2001	2021	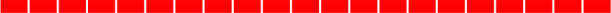
surfactant protein b	5.95	2001	2021	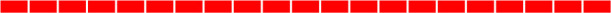
deficient mice	5.23	2001	2021	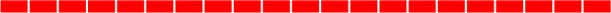
icell	5.19	2005	2021	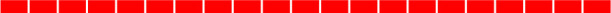
bronchoalveolar lavage fluid	5.15	2004	2021	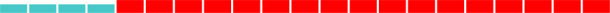
lung surfactant	5.08	2001	2021	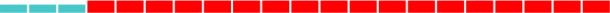
newborn	4.55	2005	2021	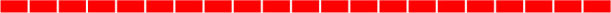
alveolar macrophage	4.23	2004	2021	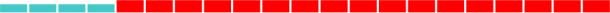
innate immunity	4.04	2006	2021	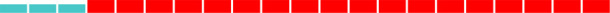
respiratory failure	3.7	2008	2021	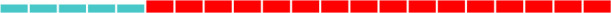
common beta chain	3.49	2008	2021	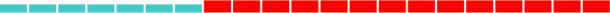
lung disease	3.43	2004	2021	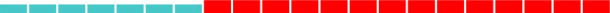
gm csf therapy	3.43	2004	2021	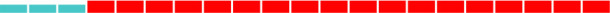
hermansky pudlak syndrome	3.43	2004	2021	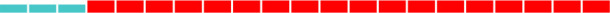
gene expression	3.33	2005	2021	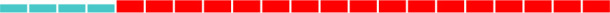
inhalation	3.31	2011	2021	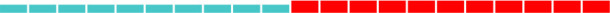
spc	3.3	2001	2021	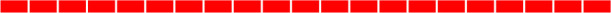
macrophage differentiation	3.18	2008	2021	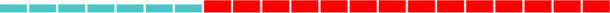

**Table 8:** The strongest citation bursts keywords after 2016.

**Keywords**	**Strength**	**Begin**	**End**	**2016–2021**
inflammation	3.83	2016	2021	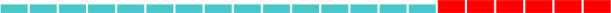
deficiency	4.41	2017	2021	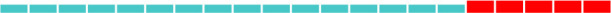
tissue resident macrophage	4.06	2017	2021	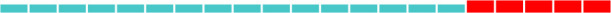
classification	3.51	2017	2021	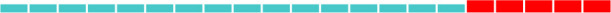
autoimmune pulmonary alveolar proteinosis	3.51	2017	2021	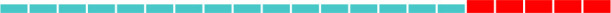
sarcoidosis	3.45	2017	2021	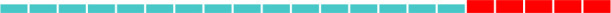
gm csf	4.06	2018	2021	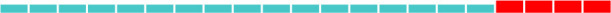
high resolution ct	3.77	2018	2021	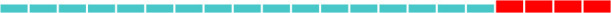
fetal monocyte	3.5	2018	2021	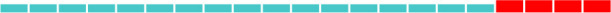

## Discussion

### General Information

Research on PAP can be roughly divided into three time periods during 2001–2021. The early-stage (2001–2009) has been increasing steadily. From 2010 to 2016, publication outputs began to increase rapidly, with three times more published in 2016 than in 2001. However, decreased publications were seen in the past 5 years but remained at a high level. This reminds us that although significant advances in our understanding of PAP over the past several decades, many important questions remain unanswered and research on PAP has still attracted great attention from scholars.

Using visual analysis of the contribution of countries and institutions, we can see that the USA, Japan, and Germany are the leading countries where PAP research is occurring. All the top 5 institutions were in these three countries. Centrality is an indicator to measure the importance of nodes in network analysis. It mainly quantifies the node's value acts as a bridge in the entire network structure. Generally, centrality values >0.1 are regarded as relatively essential nodes. As shown in Table 1, Switzerland has the highest centrality (0.55), implying it plays a pivotal role as a bridge in the worldwide international cooperation network. Nevertheless, from Figures 3, 4, the distribution of countries and institutions is scattered. The breadth and intensity of leading countries' collaboration were not ideal. For instance, there is a lack of academic cooperation between China and the USA. As for institutions, most collaborating institutions are limited to domestic ones, and there is relatively less international cooperation and exchange of findings. Considering this disease is very rare, this situation greatly hinders the development of the research field. Therefore, it is strongly recommended that institutions worldwide remove academic barriers and enhance cooperation to boost the development of PAP research.

From the perspective of authors and co-cited authors, Koh Nakata (129, 8.6%) contributed the most, followed by Bruce C Trapnell (114, 7.6%), Ryushi Tazawa (78, 5.2%), Brenna Carey (69, 4.6%), and Takuji Suzuki (65, 4.3%). It is noteworthy that Bruce C Trapnell (0.19) exerts a significant publication impact and has made the most outstanding contributions in the field of PAP during the last 20 years. Dr. Trapnell is Professor of Medicine and Pediatrics at the University of Cincinnati and an attending physician at Cincinnati Children's Hospital Medical Centers. He focused on the pathogenesis and therapy of rare lung diseases, including PAP. In 2007, Dr. Trapnell and Kanji Uchida et al. identified that GM-CSF autoantibodies caused autoimmune PAP (aPAP) and initiated clinical laboratory tests for its diagnosis (26). Since 2010, Dr. Trapnell and Takuji Suzuki et al. found hereditary PAP (hPAP) as a new genetic disease due to mutations in CSF2RA and CSF2RB (22, 27, 28). In 2014, his research group developed pulmonary macrophage transplantation (PMT), an innovative type of cell transplantation, and is currently translating it as the first specific therapy in patients with hereditary PAP (29). In 2018, Dr. Trapnell and Cormac McCarthy found that the GM-CSF signaling abnormalities lead to AMs dysfunction, including its ability to process and clear out cholesterol. They creatively use cholesterol-busting statins as a novel pathogenesis-based pharmacotherapy of PAP (30). Among the top 5 co-cited authors, Takayuki Kitamura (357) and John F. Seymour (290) established that idiopathic PAP, aka autoimmune PAP, neutralizes GM-CSF in the early research period (17). Therefore, based on this finding, a novel serological diagnosis is proposed (31).

According to the journals and co-cited journals in Table 4, the *American Journal of Respiratory and Critical Care Medicine* had the highest number of publications and citations. Almost all the top 5 co-cited journals belong to Q1. It can be seen that the research regarding etiology, pathogenesis, current and emerging therapies, and management is a hot topic presently and also a future development trend. The analysis of the literature sources is helpful to find the core journals in the research field. It can be seen that the cited articles are all from high-impact journals, indicating that a study on PAP is considered of high value by academics worldwide.

As shown in Table 5, the most frequently cited article was published by Bruce C Trapnell in *NEJM* in 2003 (15), which reports that GM-CSF autoantibodies are markedly elevated in autoimmune PAP but not in patients with secondary PAP, congenital PAP, other lung diseases, or healthy people. This finding considerably changed our concepts of the pathogenesis and treatment of PAP.

### The Hotspots and Frontiers

Keyword co-occurrence analysis focused on understanding the distribution and development of research hotspots in a particular field. As shown in Table 6, pulmonary alveolar proteinosis (522), colony-stimulating factor (285), gm-csf (121), surfactant (32), whole lung lavage (25), macrophages (33), and mutations (33) are keywords with high occurrence frequency. Cluster analysis was performed based on keywords. Eventually, four colors clusters were generated. Based on these two analyses, the research hotspots and development frontiers in the pulmonary alveolar proteinosis research field are as follows:

#### Pathogenesis and Classification of PAP

Pulmonary alveolar proteinosis (PAP) results from abnormalities of pulmonary surfactant homeostasis, usually owing to AM dysfunction. Attenuated AM maturation is generally caused by insufficient granulocyte-macrophage colony-stimulating factor (GM-CSF) signaling, which is essential for the development of AMs metabolic and immune functions (34). Autoimmune PAP, formerly known as idiopathic PAP, is the best-studied PAP-causing disease. It occurs when elevated levels of GM-CSF autoantibodies lead to the shortage of bioavailable GM-CSF. Congenital PAP (cPAP) or hereditary PAP, occurs when genetic defects of GM-CSF receptor α or β chains (*CSF2RA, CSF2RB*) lead to impaired AMs differentiation (27). Finally, secondary PAP (sPAP) results from AMs dysfunction due to hematopoietic disorders, immune dysregulation, environmental exposures, and pharmaceutical agents (16). In some rare cases, the etiology of PAP remains uncertain and the patient is diagnosed with unclassified PAP. Autoimmune PAP comprises the biggest share (90–95%) of adult patients, whereas secondary PAP accounts for 5–10% of adult cases (19).

#### Role of GM-CSF in PAP

As a 23 kDa glycoprotein cytokine produced by type II alveolar epithelial cells, GM-CSF is named for its capacity for stimulating the formation of neutrophil and macrophage colonies (35). It plays a pivotal role in the terminal differentiation of AMs. GM-CSF signaling via transcription factor PU.1 (36) and peroxisome proliferator-activated receptor-γ (PPARγ) (37, 38) is essential for the functions of AMs, including cholesterol export, surfactant clearance, immunity, and others (39). It means that GM-CSF signaling links with cholesterol homeostasis in the lungs.

The pathogenesis of PAP remained obscure until the fortuitous discovery that GM-CSF knock-out mice developed pulmonary surfactants accumulation, remarkably identical to phenotype to human PAP (18, 20). Moreover, knock-out mice deficient in the GM-CSF receptor β-chain (*Csf2rb*^−/−^ mice) (40, 41) or GM-CSF receptor α-chain (*Csf2ra*^−/−^ mice) (42, 43) also developed a lung phenotype similar to PAP caused by *CSF2RA* or *CSF2RB* mutations observed in children. They also had reduced expression of PPARγ and PU.1, which resulted in cholesterol accumulation within AMs, leading to a frothy appearance of macrophages and a decline in the uptake and clearance of surfactant (6, 39). GM-CSF augmentation was also found to correct the alveolar proteinosis in GM-CSF-deficient mice. These and further animal observations established the critical part played by GM-CSF in the proper functioning of human AMs (37). Additionally, it indicates that GM-CSF controls cholesterol efflux in a constitutive, reversible, and dose-dependent style (39).

Neutralizing GM-CSF autoantibodies are observed at high levels in autoimmune PAP patients (17, 34) but not in those with congenital or secondary PAP, other respiratory diseases, or healthy people (21, 44). Furthermore, injection of GM-CSF autoantibodies derived from patients with autoimmune PAP into healthy non-human primates reproduced the characteristics of PAP (45, 46), thus proving the hypothesis that autoimmune PAP results from an autoantibodies-mediated disorder of GM-CSF signaling.

#### Treatment of PAP

##### Therapeutic whole-lung lavage

Since its initial description in 1964, WLL has been the forefront therapy for PAP (but not congenital PAP) (47). In brief, it is a single or sequential bilateral lavage accompanied by isolation of the two lungs using a double-lumen endotracheal tube under general anesthesia (48). Normal saline is instilled into the lung, then the milky and opaque effluent is drained repeatedly until the effluent becomes clear (49). Segmental lavage has also been done through bronchoscopy (bronchoalveolar lavage) in some medical centers. Therapeutic efficacy derives from removing the excessive surfactant by physically “washing” the alveoli with saline (50). Although WLL is generally regarded as a safe procedure that could improve symptoms, radiographic abnormalities, and oxygenation in patients, it is not without complications, including hypoxia, pneumothorax, hydrothorax, infection, and acute respiratory distress syndrome (3). Moreover, the procedure has not yet been standardized remains highly operator-dependent. In conclusion, WLL, notwithstanding being an invasive procedure, remains the cornerstone of therapy for moderate to severe PAP.

#### Emerging Pathogenesis-Based Therapies

##### GM-CSF therapy

The discovery that insufficient GM-CSF bioavailability is the pathogenesis of autoimmune PAP aroused interest in the therapeutic use of GM-CSF. In 1996, a patient received recombinant human GM-CSF (rhGM-CSF) by subcutaneous administration for the first time, resulting in a significant improvement in symptoms and oxygenation (51). Since then, several other cohort studies using subcutaneous delivery have reported similar findings with objective improvements in ~50% of cases and accumulated plenty of available efficacy data (33, 52).

Previously, aerosolized GM-CSF has been the most promising therapy in autoimmune PAP. In a prospective trial conducted in Japan (25), 35 Japanese patients with autoimmune PAP inhaled GM-CSF over a total period of 24 weeks, using a regimen of initial high-dose followed by a maintenance low-dose. 62% of patients demonstrated an improvement in both subjective (dyspnoea) and objective parameters (6-minute walk test), whereas serum GM-CSF autoantibody levels remained static. After an extended follow-up of 30 months, 66% of patients require no further additional treatments. Most importantly, unlike the subcutaneous administration, no significant side-effects have been observed in any of the trials of inhaled GM-CSF. In a recent randomized, controlled trial, inhaled recombinant human GM-CSF (sargramostim) was associated with a modest salutary effect on the laboratory outcome of arterial oxygen tension, and no clinical benefits were noted (24). In 2020, a double-blind, placebo-controlled, three-group trial assigned patients with autoimmune PAP to receive the recombinant GM-CSF molgramostim (300 μg once daily by inhalation), either continuously or intermittently (every other week), or matching placebo. For multiple end points, improvement was greater with continuous molgramostim than with intermittent molgramostim or placebo (53). Overall, clinical trials show that inhaled GM-CSF in patients with autoimmune PAP is safe and effective. However, the optimal dose, timing, or duration of administration should be further defined for response rate improvement.

##### Therapy Targeting GM-CSF Autoantibodies

Since the finding of the pathogenesis of GM-CSF autoantibodies, different therapeutic strategies targeting a lower level of the autoantibodies have been adopted in autoimmune PAP. By analogy with other autoimmune diseases, corticosteroids seem reasonable to treat autoimmune PAP. However, results from PAP patients treated with corticosteroids show more harm than good (54). Other therapies include rituximab (a monoclonal anti-CD20 antibody) to remove B lymphocyte and autoantibodies depletion using plasmapheresis. Plasma exchange has been used with marginal success, and the results have not been consistent enough for a recommendation (55, 56). As for rituximab, one open-label trial demonstrated improvement in A-aDO_2_ gradient in seven out of the nine patients completing the study (23); A retrospective study showed that no patient had marked improvement after 6-month treatment (32). Further prospective studies are demanded before the utility of those conceptually feasible therapies.

##### Pulmonary Macrophage Transplantation

Hereditary PAP deficient in GM-CSF receptor, thus, other therapeutic options are needed. Animal studies showed that bone marrow transplantation (BMT) can re-establish surfactant homeostasis and correct hereditary PAP. Although BMT had minimal success in hereditary PAP children, it is restricted by the morbidity and mortality of myeloablation, and secondary PAP can itself be a complication of BMT (57). Pulmonary macrophage transplantation (PMT) is a novel cell transplantation method that has demonstrated therapeutic efficacy in animal studies (29, 42, 43). These reports support the translation of PMT as a specific therapy for children with hereditary PAP.

##### Targeting Pulmonary Cholesterol Homeostasis

GM-CSF signaling interruption reduced cholesterol clearance from AMs, which is the leading pathogenesis of PAP. This led to the consideration of therapy targeting cholesterol homeostasis as an alternative option for PAP. PPARγ agonist therapy escalated cholesterol clearance and reduced PAP disease severity of knock-out mice (39). This discovery has translated to a clinical trial of pioglitazone (a PPARγ agonist). In addition, statin is associated with decreased cholesterol accumulation of AMs and PAP disease remission. As for patients with autoimmune PAP, statin therapy ameliorates PAP significantly (30), supporting the possibility of statins as innovative pathogenesis-based therapies.

## Conclusions

This study summarizes the research status of PAP in the past 20 years. Publications related to PAP are increasing over time. Different countries/regions and institutions need to deepen and strengthen cooperation. The majority of the articles regarding PAP are published in and cited from influential international journals, suggesting that PAP has attributed much attention. Several cardinal questions remain unanswered, such as the etiology of autoimmune PAP and the pathogenesis of secondary PAP. Those critical issues need to be put on the front burner. This study provides assistance for scholars to find core literature and partners in PAP, contributes direction for journals publication, and guidelines identifying research hotspots in this field.

## Data Availability Statement

The original contributions presented in the study are included in the article/supplementary material, further inquiries can be directed to the corresponding author.

## Author Contributions

SL and GL conceived and designed the study. SL, KX, and JH collected the data. DW and XY re-examined the data. JZ and XL analyzed the data. SL wrote the first draft of the manuscript. LB and KX wrote sections of the manuscript. XC reviewed and revised the manuscript. All authors contributed to the article and approved the submitted version.

## Funding

This work was supported by the National Natural Science Foundation of China (Grant No. 82074262).

## Conflict of Interest

The authors declare that the research was conducted in the absence of any commercial or financial relationships that could be construed as a potential conflict of interest.

## Publisher's Note

All claims expressed in this article are solely those of the authors and do not necessarily represent those of their affiliated organizations, or those of the publisher, the editors and the reviewers. Any product that may be evaluated in this article, or claim that may be made by its manufacturer, is not guaranteed or endorsed by the publisher.
